# Palliative Care Consult for Clinician Distress Through the Philosophical Lenses of Gender Norms and Phenomenology

**DOI:** 10.1093/geroni/igaa057.790

**Published:** 2020-12-16

**Authors:** Anessa Foxwell, Salimah Meghani, Connie Ulrich

**Affiliations:** 1 University of Pennsylvania, Philadelphia, Pennsylvania, United States; 2 University of Pennsylvania School of Nursing, Philadelphia, Pennsylvania, United States

## Abstract

The National Academy of Medicine has raised significant concerns on clinician health and well-being as many experiencing burnout, post-traumatic stress, and depression. Indeed, clinicians experience a range of human emotions when caring for older adults with severe, life-limiting illnesses. These emotions may manifest in multiple ways and from various sources. Uncertain of how to attend to such distress, clinicians may consult a trusted resource, including the palliative care team. Palliative care specialists are trained to support the complexities and needs of patients and families; increasingly, however, palliative care consults are rooted in clinician distress. This session uses clinical case examples to explore the palliative care consult for distressed clinicians from two different philosophical perspectives: (1) phenomenology and (2) the social construct of gender norms. A phenomenological lens respects the unique, subjective lived experience of each individual in their day-to-day interactions with patients, families, and health care systems. Therefore, when caring for seriously ill older adults, clinicians may bring their own subjective experiences to the patient encounter and react differently to ethical dilemmas and conflicts that arise. The social construct of gender norms asks us to examine clinician distress from a different perspective. Here, the postmodern rejection of gender binarism allows clinicians to experience a spectrum of emotions and distress regardless of gender. Exploration through clinical cases will highlight the unique, varied experience of clinician distress and offer opportunities for future research into the role of palliative care teams in supporting distressed clinicians who care for seriously ill older adults.

